# Supplementary material for: Protocol for an extended scoping review on the use of virtual nominal group technique in research
Source: PLoS One. 2023 Jan 20;18(1):e0280764. doi: 10.1371/journal.pone.0280764 (PMC9858029; doi:10.1371/journal.pone.0280764)
Supplement: S2 File — (DOCX) [file pone.0280764.s002.docx]

**APPENDIX 2 June 28, 2022**

**Search Methods**

Search strategies were developed by an information specialist (KF) and peer reviewed using the PRESS guideline (1). The searches were conducted June 28^th^, 2022 in: MEDLINE(R) ALL (OvidSP), Embase (OvidSP), CINAHL (EBSCOHost), ERIC (OvidSP), Education Source (EBSCOHost), APA PsycInfo (OvidSP), Web of Science, and Scopus. Each database was search from its inception.

**RESULTS**

The electronic search of the databases identified 11,452 citations. 7,364 duplicate records were removed using Covidence (Veritas Health Information, Melbourne, Australia), which left 4,116 references for the screening phase.

**Acknowledgements**

We thank Sarah Visintini, MLIS (Research Librarian, University of Ottawa Heart Institute) for peer review of the MEDLINE search strategy.

**References**

1. McGowan J, Sampson M, Salzwedel DM, Cogo E, Foerster V, Lefebvre C. PRESS Peer Review of Electronic Search Strategies: 2015 Guideline Statement. J Clin Epidemiol. 2016 Jul;75:40–6.
2. Harb SI, Tao L, Peláez S, Boruff J, Rice DB, Shrier I. Methodological options of the nominal group technique for survey item elicitation in health research: A scoping review. *Journal of Clinical Epidemiology*. 2021;139:140-148. doi:[10.1016/j.jclinepi.2021.08.008](https://doi.org/10.1016/j.jclinepi.2021.08.008)

**Supplemental Files**

| Ovid **MEDLINE(R) ALL** <1946 to June 27, 2022> | | |
| --- | --- | --- |
|  |  |  |
| 1 | (nominal adj2 (group* or consensus)).ti,ab,kf. | 1999 |

| **Embase** <1947 to 2022 June 27> | | |
| --- | --- | --- |
|  |  |  |
| 1 | (nominal adj2 (group* or consensus)).ti,ab,kf. | 2660 |
| 2 | Conference Abstract.pt. | 4434090 |
| 3 | 1 not 2 | 1988 |

| APA **PsycInfo** <1806 to June Week 3 2022> | | |
| --- | --- | --- |
|  |  |  |
| 1 | (nominal adj2 (group* or consensus)).tw. | 682 |

| **ERIC** <1965 to April 2022> | | |
| --- | --- | --- |
|  |  |  |
| 1 | (nominal adj2 (group* or consensus)).tw. | 291 |

## **CINAHL** (EBSCOHost)

| \| **#** \| **Query** \| **Results** \| \| --- \| --- \| --- \| \| S1 \| TI ( nominal N2 (group* or consensus) ) OR AB ( nominal N2 (group* or consensus) ) \| 1,086 \| |
| --- | --- | --- | --- | --- | --- | --- |

## **Education Source** (EBSCOHost)

| \| **#** \| **Query** \| **Results** \| \| --- \| --- \| --- \| \| S1 \| TI ( nominal N2 (group* or consensus) ) OR AB ( nominal N2 (group* or consensus) ) OR KW ( nominal N2 (group* or consensus) ) \| 335 \| |
| --- | --- | --- | --- | --- | --- | --- |

**Scopus**

( TITLE ( "nominal group*" OR "nominal consensus" ) OR ABS ( "nominal group*" OR "nominal consensus" ) )

Results: 2,772

**Web of Science**

"nominal group*" OR "nominal consensus" (Title) or "nominal group*" OR "nominal consensus" (Abstract) and Meeting Abstracts or Book Reviews or News Items or Retracted Publications (Exclude – Document Types)

Results: 2,299
