# Supplementary material for: Protocol for an extended scoping review on the use of virtual nominal group technique in research
Source: PLoS One. 2023 Jan 20;18(1):e0280764. doi: 10.1371/journal.pone.0280764 (PMC9858029; doi:10.1371/journal.pone.0280764)
Supplement: S3 File — (DOCX) [file pone.0280764.s003.docx]

**APPENDIX 3**

Searches performed July 15^th^, 2022.

The electronic search of the databases identified 2,589 citations. 1,656 duplicate records were removed using Covidence (Veritas Health Information, Melbourne, Australia), which left 933 references for the screening phase.

**Supplemental Files**

| Ovid MEDLINE(R) ALL <1946 to July 14, 2022> | | |
| --- | --- | --- |
|  |  |  |
| 1 | (nominal adj2 (group* or consensus)).ti,ab,kf. | 2011 |
| 2 | limit 1 to yr="2020 -Current" | 529 |

| Embase <1947 to 2022 July 14> | | |
| --- | --- | --- |
|  |  |  |
| 1 | (nominal adj2 (group* or consensus)).ti,ab,kf. | 2676 |
| 2 | limit 1 to yr="2020 -Current" | 582 |

| APA PsycInfo <1806 to July Week 2 2022> | | |
| --- | --- | --- |
|  |  |  |
| 1 | (nominal adj2 (group* or consensus)).tw. | 683 |
| 2 | limit 1 to yr="2020 -Current" | 64 |

| ERIC <1965 to June 2022> | | |
| --- | --- | --- |
|  |  |  |
| 1 | (nominal adj2 (group* or consensus)).tw. | 291 |
| 2 | limit 1 to yr="2020 -Current" | 6 |

## **CINAHL** (EBSCOHost)

| \| **#** \| **Query** \| **Limiters/Expanders** \| **Results** \| \| --- \| --- \| --- \| --- \| \| S1 \| TI ( nominal N2 (group* or consensus) ) OR AB ( nominal N2 (group* or consensus) ) OR KW ( nominal N2 (group* or consensus) ) \| Expanders - Apply related words; Apply equivalent subjects  Search modes - Boolean/Phrase \| 1,091 \| \| S2 \| TI ( nominal N2 (group* or consensus) ) OR AB ( nominal N2 (group* or consensus) ) OR KW ( nominal N2 (group* or consensus) ) \| Limiters - Published Date: 20200101-20221231  Expanders - Apply related words; Apply equivalent subjects  Search modes - Boolean/Phrase \| 207 \| |
| --- | --- | --- | --- | --- | --- | --- | --- | --- | --- | --- | --- | --- |

## **Education Source** (EBSCOHost)

| \| **#** \| **Query** \| **Limiters/Expanders** \| **Results** \| \| --- \| --- \| --- \| --- \| \| S2 \| TI ( nominal N2 (group* or consensus) ) OR AB ( nominal N2 (group* or consensus) ) OR KW ( nominal N2 (group* or consensus) ) \| Limiters - Published Date: 20200101-20221231  Expanders - Apply related words; Apply equivalent subjects  Search modes - Boolean/Phrase \| 35 \| \| S1 \| TI ( nominal N2 (group* or consensus) ) OR AB ( nominal N2 (group* or consensus) ) OR KW ( nominal N2 (group* or consensus) ) \| Expanders - Apply related words; Apply equivalent subjects  Search modes - Boolean/Phrase \| 336 \| |
| --- | --- | --- | --- | --- | --- | --- | --- | --- | --- | --- | --- | --- |

**Scopus**

( TITLE ( "nominal group*" OR "nominal consensus" ) OR ABS ( "nominal group*" OR "nominal consensus" ) ) AND ( LIMIT-TO ( PUBYEAR , 2022 ) OR LIMIT-TO ( PUBYEAR , 2021 ) OR LIMIT-TO ( PUBYEAR , 2020 ) )

Results: 610 references retrieved

**Web of Science**

"nominal group*" OR "nominal consensus" (Title) or "nominal group*" OR "nominal consensus" (Abstract)

Refined: Publication years: 2020, 2021, 20222

Results: 556 references retrieved
