# Supplementary material for: Protocol for an extended scoping review on the use of virtual nominal group technique in research
Source: PLoS One. 2023 Jan 20;18(1):e0280764. doi: 10.1371/journal.pone.0280764 (PMC9858029; doi:10.1371/journal.pone.0280764)
Supplement: S4 File — (DOCX) [file pone.0280764.s004.docx]

**APPENDIX 4**

Online survey of corresponding authors for included studies

1. Which virtual platform did you use?

[ ] Google Hangouts

[ ] Microsoft Teams

[ ] Skype

[ ] Zoom

[ ] Other (please specify): _______

2. For each step of the Nominal Group Technique (NGT) please list if you used a virtual platform or Face to face

Idea generation

[ ] Virtual

[ ] Face-to Face

[ ] other (please specify): ______

Sharing of ideas (usually round robin)

[ ] Virtual

[ ] Face-to Face

[ ] other (please specify): ______

Discussion/clarification

[ ] Virtual

[ ] Face-to Face

[ ] other (please specify): ______

Voting

[ ] Virtual (specify if different than above) ________

[ ] Face-to Face

[ ] other (please specify): ______

3. Which of the following functions did you use?

[ ] Annotations

[ ] Chat

[ ] Polls

[ ] Other (please specify): _______

4. What modifications did you make to the NGT, if any, to accommodate the virtual format?

[ ] none

[ ] other (please specify) __________________

5. Why did you use a virtual format for your Nominal Group Technique? (select as many as apply)

[ ] COVID restrictions for face-to-face meetings

[ ] COVID travel restrictions

[ ] Decision had nothing to do with COVID

[ ] Allows for participants from different geographic regions

[ ] Other (please specify) ____________________

How many NGT have you participated in (moderator or participant) that were Face-to-Face

How many NGT have you participated in (moderator or participant) that were not Face-to-Face?

6. Overall, what are your general impressions of how the virtual platform worked?

[ ] overall performed similarly to face-to-face

[ ] overall better than face-to-face

[ ] overall not as good as face-to-face

Comments _____________________________________________________________

7. What were the benefits of using a virtual NGT?

[ ] save money

[ ] save travel time and expense

[ ] allowed for increased choice when selecting of participants

[ ] other _______________________________________________________________

8. What were challenges encountered and how did you manage these?

[ ] participants not familiar with platform/technology

[ ] technology issues

[ ] reduced participant engagement

[ ] challenges moderating

[ ] other:

Comments

_____________________________________________________________

9. Have you written a methods or reflections paper based on your study? If so, what is the

relevant citation information?

10. If you were to compare your experience with face to face compared to the virtual NGT what would you consider are the most important differences?

________________________________________________________________________

11. If someone were new to the virtual NGT, what suggestions or lessons learned would you share with them?

_______________________________________________________________________

12. Would you be willing to be contacted in the future for a focus group to consider the pros and cons of the virtual NGT?

[ ] yes

[ ] no

Thank you for completing the survey!
