# Supplementary material for: Protocol for an extended scoping review on the use of virtual nominal group technique in research
Source: PLoS One. 2023 Jan 20;18(1):e0280764. doi: 10.1371/journal.pone.0280764 (PMC9858029; doi:10.1371/journal.pone.0280764)
Supplement: Attachment — Submitted filename: Response to Reviewers .docx [file pone.0280764.s006.docx]

Ernesto Iadanza

Academic Editor

PLOS ONE

Dear Dr. Ernesto Iadanza,

RE: PONE-D-22-27748

Protocol for an Extended Scoping Review on the use of Virtual Nominal Group Technique in Research

Thank you for providing us an opportunity to re-submit our manuscript. As part of the re-submission, please note our error in the authorship order, it has been revised. The response to reviewers has been uploaded as a separate word document titled “Revisions table response to reviewers”.

Regarding the other items:

1. Style has been verified to match the PLOS one requirements. Please note that the Supplemental Files have been re-uploaded with the correct PLOS one title requirements as well.

2. We have updated the grant information (our local grants do not have “grant numbers” so not included). The financial disclosure now matches the funding information.

3. Upon reflection we have changed our response to the repository information for data to meet requirements of our ethics board. “The entirety of the data collected and analyzed for this review will be within the manuscript and its Supporting Information files. Interview data will be made available upon request and approval through the University of Ottawa Research Ethics board.”

4. Full ethics statement provided in the manuscript under methods.

5.Uploaded copies of the marked-up manuscript with track changes titled “Revised Manuscript with Track Changes” and an unmarked version of the manuscript titled “Manuscript”. Please note that this manuscript is a scoping review and two PRISMA Checklists have been uploaded as Supplemental Files (S1_PRISMA SCR Checklist and S5_PRISMA-P Checklist)

6. The reference list has been analyzed and follows PLOS one’s referencing guidelines.

We look forward to your response.

Sincerely,

Susan Humphrey-Murto

on behalf of the research team
